# An automated platform for “on-demand” high-speed catalyst synthesis by flame spray pyrolysis

**DOI:** 10.1039/d5dd00042d

**Published:** 2025-10-10

**Authors:** Konstantin M. Engel, Patrik O. Willi, Robert N. Grass, Wendelin J. Stark

**Affiliations:** a Functional Materials Laboratory, Institute for Chemical and Bioengineering, ETH Zürich Vladimir-Prelog-Weg 1/1-5 8093 Zürich Switzerland wendelin.stark@chem.ethz.ch

## Abstract

Flame-Spray Pyrolysis (FSP) is a versatile synthetic aerosol method to produce inorganic mixed-metal nanoparticles, frequently used for catalysts, battery materials, or chromophores. This work introduces a novel automated robotic platform based on FSP – AutoFSP – to accelerate materials discovery and optimization while providing standardized, machine-readable documentation of all synthesis steps. The manuscript outlines the design considerations for both hardware and software of AutoFSP, as well as the platform's performance in terms of speed, accuracy, and repeatability. AutoFSP has demonstrated significant time savings by reducing operator workload by a factor of two to three, while also improving documentation and decreasing the chance of human experimental error. AutoFSP achieves high compositional accuracy and precision across two orders of magnitude. The relative error of the effective molar metal loading *x* in Zn_*x*_Zr_1−*x*_O_*y*_ and In_*x*_Zr_1−*x*_O_*y*_ nanoparticles produced with the setup remains within ± 5%. The platform showcases the potential of automation in chemical discovery and exemplifies how established manual synthetic methods can be adapted for robotic processes before integration into a materials acceleration platform (MAP).

## Abstract 2

This work presents AutoFSP, an automated platform for the high-speed synthesis of nanoparticles by flame spray pyrolysis (FSP).

## Graphical abstract

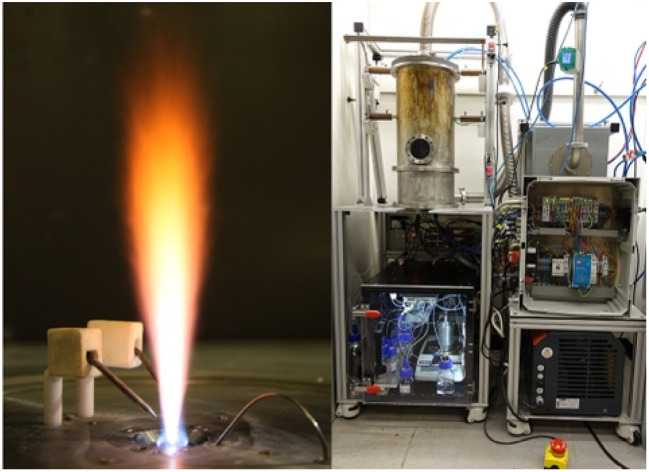

## 1. Introduction

Catalyst research and process development are tightly interlinked and cover phenomena over 6 to 14 orders of magnitude in size, mass, speed, and energy.^1^ To reduce the risk of a “late drop-out catalyst” as early as possible in the scale-up process, it would be advantageous to employ high-throughput synthetic methods that provide a high flexibility concerning batch size, composition, and physical properties of the target materials.^2,3^ Such synthetic methods can significantly support the understanding of heat and mass transfer of such systems at both pilot and production scale, by providing the relevant materials on short notice. A systematic performance screening of potential catalyst compositions calls for rapid and time-efficient access to complex inorganic materials.^4^ Ideally, these materials should possess well-defined, yet tunable physical properties. Furthermore, a wide array of compositions should be available at a minimum requirement for adjustments to the synthetic protocol.

Compared to traditional methods like incipient wetness impregnation and co-precipitation, Flame Spray Pyrolysis (FSP) has proven to be a powerful and highly versatile synthetic approach. It can be used to synthesize high-temperature, inorganic, pure, or mixed metal oxide (MMO) nanoparticles,^5,6^ that are commonly used as catalysts,^7–10^ but also in sensor applications,^11,12^ or as battery materials.^13,14^

FSP relies on several physical liquid-to-gas-to-solid steps, such as precursor evaporation, oxidation, nucleation, and subsequent solid particle growth mechanisms, resulting in highly characteristic particle architectures that may differ considerably from that of a material of the same nominal composition produced *via* wet chemistry.^8,11^

**Fig. 1:**
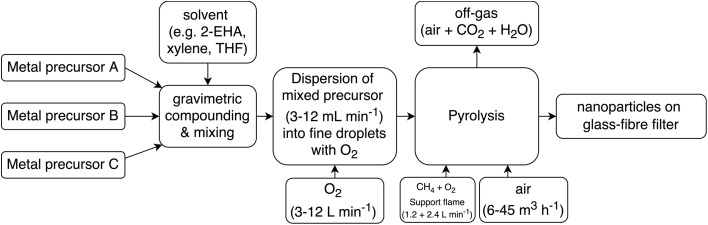
Block flow diagram of steps performed during FSP synthesis. These must be performed in both manual and automatic modes.

The individual process steps performed during an FSP synthesis are depicted in Fig. 1. The precursor is dispersed with the help of a custom-made nozzle, centered within an annular flame of CH_4_/O_2,_ which ignites the fine mist. This burner ensemble is located inside an enclosure, called a reactor. It consists of the actual body and a water-cooled lid which holds a high-temperature glass fiber filter. By allowing an airstream to flow through the reactor, the particles formed within the flame are drawn to accumulate on the top-installed filter instead of recirculating through the flame to form bigger agglomerates. By variation of the O_2_-to-fuel ratio, the residence time of the particles therein can be influenced, which in turn is used to tailor their size and structure.^15^ Furthermore, particle morphology may be modified by varying the nature of the solvent mixture.^16^

Although the product's specific surface area tends to be high (60–200 m^2^ g^−1^), the particles are relatively unsusceptible towards sintering even at elevated temperatures (*e.g.* 600 °C)^17^ where thermal mobility is high, and reduction of specific surface areas would occur readily on materials prepared by a comparable low-temperature method (*i.e.*, co-precipitation). The two production parameters that can be easily varied in FSP are particle composition (mixing unit) and particle size (flame conditions). Batch sizes are simple to scale up, and in principle, the process can be operated continuously.^18,19^

Commonly, the widely commercially available metal salts of 2-ethylhexanoic acid (2-EHA) are used as precursors in FSP.^5^ Due to their good miscibility and air stability, they can be flexibly used in precursor mixtures with varying ratios of the respective metals, without affecting flame quality. Their low prices and good commercial availability are essential factors to be considered for easy scale-up if a successful candidate is found.

In catalyst discovery and optimization, a commonly used approach is to vary the composition of a knowingly well-performing MMO system,^20^ systematically screening different elements for doping while keeping the primary component unchanged.^10,21^ Additionally, the total number of components may be increased to create complex multi-phase tertiary^8,10,22^ and potentially even quaternary materials.^23^ Using FSP, such MMO nanoparticles can be produced by thoroughly mixing the liquid precursors in the desired target molar ratio and pyrolyzing this mixture under highly oxidizing conditions. Depending on the molar ratios of the elements and the chemical nature of their mixtures, the less abundant element may appear to be homogeneously distributed throughout the oxide matrix like a solution or form larger clusters if this is energetically favored or the loading is sufficiently high. To what degree “mixing” or “unmixing” occurs on a nanometer level is hardly predictable and is defined by the complex processes within the flame, during particle nucleation and growth, and the cooling trajectory. With few exceptions, it can generally be assumed that the molar ratio of elements in the precursor directly translates to the same ratio in the mixed metal oxide. Furthermore, besides oxidic particles, halides^24^ or phosphates with varying M-to-PO_4_ ratios^25^ can be made with FSP under oxidizing conditions.

The resulting nanopowders typically possess similar specific surface areas regardless of their composition. If needed, this parameter can be fine-tuned by adjusting the flame conditions. On the other hand, such screening approaches require many repetitions of very similar tasks. These tend to be error-prone, while taking up laboratory resources.

Automation encompasses a partial or complete elimination of human intervention.^26^ It has furthered the field of catalyst research in the recent past, as for example, the development and commercialization of automated setups for synthesis *via* impregnation or (co-)precipitation by Chemspeed Technologies AG.^27,28^ However, these methods have the downside of relying on difficult powder handling, making synthesis error-prone and harder to replicate. On the other hand, FSP uses liquids, which typically simplifies handling steps. Although automation could provide significant opportunities for the FSP technique, there seems to be no highly automated setup available for systematically screening flame-made MMOs. To our knowledge, the only setup that offers basic automation without any mixing function or advanced process control is NPS-20 by ParteQ GmbH.^29^

### 1.1 User-application-oriented approach for automating FSP

The design of such a novel automated FSP platform should fulfil the needs and specifications set by its current users, while also anticipating future use cases and successful integration into comprehensive autonomous workflows within the framework of materials acceleration platforms (MAPs).^30^ A survey by Hung *et al.*^31^ investigated motivations and challenges related to laboratory automation. It defines five broad categories of automation: process execution, data analysis, data interpretation, decision making, and communication in workflows. The survey points out that experimentalists' primary motivation for automation is improving efficiency. When applying these requirements to FSP, the focus of automation should be on executing the process quickly, reliably, and reproducibly, yet safely. Furthermore, standardization of input and output communication is an essential preliminary for inclusion of such a setup into a MAP.

In this manuscript, we present a novel, automated FSP platform, which we named AutoFSP. As it performs most steps required for materials synthesis with minimal user intervention, it could pave the way to integration of FSP into a more comprehensive MAP operating at a level of autonomy, L1, or higher, as defined by Hung *et al.*^31^

To our knowledge, no robotic FSP system that operates on a comparable level of automation exists. The description of the instrument and the validation of its performance are provided in the following sections. The SI and associated data repository^32^ contain the PLC-code, templates for the csv-files used for data submission to and from the instrument, machining instructions for custom parts and a bill of materials including estimated pricing of all items required to replicate the build.

For the preparation of the manuscript, we aimed to follow the guidelines established by the editorial board for hardware-focused articles.^33^

## 2. Experimental methods

### 2.1 Programmatic and physical conception of system control

Operation of AutoFSP is achieved using a programmable logic controller (PLC, PFC100, WAGO, Minden, Germany) along with the corresponding input–output modules for connecting sensors and actuators. The PLC-code was written in the CODESYS® based e!Cockpit® programming environment supplied by the controller's manufacturer (WAGO). CODESYS^®^ is a manufacturer-independent IEC 61131-3 automation software for engineering control systems. A 12.5′′ touch screen connected to a Raspberry Pi 4 serves as Human Machine Interface (HMI), accessing the PLC visualizations by means of a standard browser application. A comprehensive list of the hardware components and the code required for AutoFSP's operation can be found in the Zenodo data repository.

### 2.2 Preparation of InZrO_*x*_ and ZnZrO_*x*_ by AutoFSP

#### 2.2.1 Synthesis of FSP precursors

All dilutions, unless otherwise specified, were performed with a 2 : 1 (w/w) mixture of 2-ethylhexanoic acid (2-EHA, Acros Organics, 99%) and tetrahydrofuran (THF, Merck, for chromatography). Commercially available Zr(iv) 2-ethylhexanoate (VALIREX Zr 24, Umicore) was diluted to yield a Zr-loading of 467 mmol kg^−1^. Commercially available Zn(ii) 2-ethylhexanoate (VALIREX Zn 22.5, Umicore) was diluted to yield Zn-loadings of 506, 51, and 5 mmol kg^−1^. In(iii) 2-ethylhexanoate prepared from elemental indium (see the SI for a detailed procedure) was diluted to concentrations of 480, 48 and 5 mmol kg^−1^. Note that the unit refers to moles of solute per kilogram of solution.

#### 2.2.2 Operation of AutoFSP for build verification

**Fig. 2:**
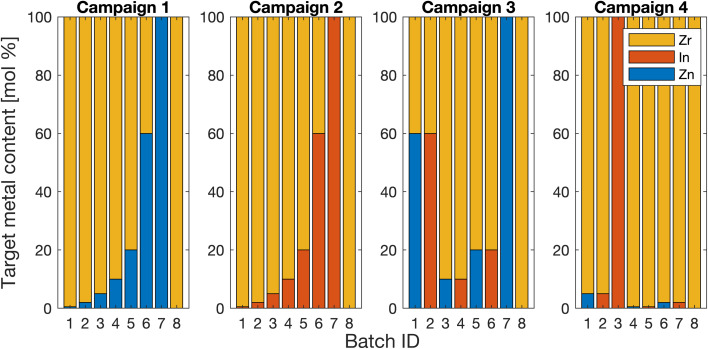
Production campaigns 1 and 2 were run to synthesize Zn_*x*_Zr_1−*x*_O_*y*_ and In_*x*_Zr_1−*x*_O_*y*_, with varying molar loadings of In on Zr and Zn on Zr, respectively. Therein, the use of Zn and In precursors was not alternated, ruling out any possible contamination. In contrast, campaigns 3 and 4 were designed to quantify batch-to-batch carryover.

A total of four production campaigns targeting Zn_*x*_Zr_1−*x*_O_*y*_ and In_*x*_Zr_1−*x*_O_*y*_ with varying compositions as depicted in Fig. 2 were performed by AutoFSP without any user intervention besides filter changes and product collection. Each campaign consisted of the sequential production of eight individual batches. The selection of Zn, In and Zr as matrix elements was driven by their relevance as catalysts and the relative ease of verifying the resulting MMO composition by ICP-OES. The investigation and rationalization of the two systems' properties and performance as catalysts are part of a separate manuscript.^34^

Campaign 1 aimed at the sequential production of eight batches of Zn_*x*_Zr_1−*x*_O_*y*_ with increasing nominal Zn loading, *x*, from 0 to 1 as defined in Fig. 2. Therein, values of *x* = 0 and *x* = 1 refer to ZrO_2_ and ZnO with no other elements added, respectively. Campaign 2 would follow the same pattern but use In and Zr to produce In_*x*_Zr_1−*x*_O_*y*_ at the same nominal compositions. Campaigns 3 and 4 aimed at a repetition of the batches from campaigns 1 and 2 while alternating the synthesis of Zn_*x*_Zr_1−*x*_O_*y*_ and In_*x*_Zr_1−*x*_O_*y*_. The selected sequence enabled investigation of carryover contamination from one batch to another.

For all campaigns, the process parameters were kept identical: compounded precursor solutions were automatically pumped into the support flame of 2.4 L per min O_2_ (99.995%, Pangas) and 1.2 L per min CH_4_ (99.9%, PanGas) through a 0.4 mm needle at a flow rate of 5.0 mL min^−1^ and dispersed into a fine spray by flowing O_2_ at 1.5 bar at a flow rate of 5 L min^−1^. Note that these volumetric gas flows refer to standard atmospheric pressure and temperature. The resulting products were collected on glass fiber filters (257 mm, GF/A-6, Hahnenmühle Life Science, Dassel, Germany) installed at the outlet of the reactor. They were manually scratched off the filter with a spatula.

Before pyrolysis, the individual batches were compounded in the dosing & mixing unit described in the next section. The automatic cleaning procedure required for this operation was run using THF (Merck, for chromatography).

### 2.3 Compositional analyses by ICP-OES

#### 2.3.1 Digestion of flame-made materials

Before compositional analysis by inductively coupled plasma-optical emission spectroscopy (ICP-OES, Horiba Ultima Expert), all flame-made powders were subjected to microwave-assisted acid digestion (Multiwave 7000, Anton Paar). To achieve complete dissolution, the digestion parameters had to be adjusted according to batch composition and can be found in the SI. To quantify the error of the analytical method (digestion and ICP-OES), each batch was analyzed in triplicate.

#### 2.3.2 Calibration of ICP-OES and batchwise compositional analysis

Analytes were diluted as to achieve values within the calibration range before ICP-OES with 1% HNO_3_ (65%, Sigma-Aldrich, for analysis, EMSURE®) in ultrapure water (MilliQ®, Merck Millipore).

From the 12 measurements performed for each loading (3 repetitions per batch, four batches with the same nominal loading), the internal (originating from the measurement) and external (originating from the synthesis) standard deviations, *σ*_int_ and *σ*_ext_, were derived. Accuracy of AutoFSP in terms of product composition was estimated through the relative standard deviation, RSD_syn_ [*σ*_ext_/mean], and the average relative deviation from the specifications, also referred to as bias, respectively, and expressed as a percentage:
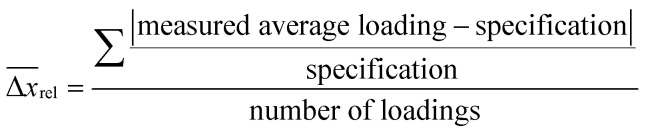


The batches that required no dosing (pure ZrO_2_, ZnO, and In_2_O_3_) were excluded from the analysis because they would bias any conclusions about dosing accuracy.

Limits of detection (LOD) and quantification (LOQ) of Zn, Zr, and In were derived from their respective calibration curves. Experimental data, along with the parameters used during calibration and measurement, can be found in the SI.

### 2.4 Characterization of specific surface area (SSA)

To further quantify the precision of AutoFSP in recreating syntheses, the specific surface areas (SSAs) of the two materials with the same nominal compositions, but issued from different campaigns, were compared on a batch-to-batch (B2B) basis. To this end, the SSAs of all materials were characterized in a physisorption apparatus (TriStar, Micromeritics, USA) using N_2_ gas and the Brunauer–Emmett–Teller (BET) method on the first five data points. Material samples (150–230 mg) were degassed at 200 °C with a specialized device (VacPrep061, Micromeritics Inc.) for three hours before measurement. The repeatability of the SSA of the physical output of AutoFSP was estimated by establishing the related relative standard deviation, RSD_AutoFSP,BET_, as outlined and discussed in the Results & discussion section.

### 2.5 Recording of X-ray diffraction patterns

The powder X-ray diffraction (XRD) patterns of a selection of batch-pairs with the same nominal composition but produced in separate campaigns were recorded using an X-ray diffractometer (X'Pert Pro, PANalytical B.V., Netherlands) with Cu-Kα as the radiation source (*λ* = 1.5406 Å). Mean crystallite size was derived using the Scherrer equation.^35–37^ The similarity in crystallography of the pairs was analyzed with the help of the Procrustes distance available through a standard command in Matlab.^38^

## 3. AutoFSP overview

The development of AutoFSP was an optimization process, which included numerous changes to the setup's software and hardware. As the mechatronic interplay of these two is the key innovation and the “heart” of AutoFSP, we will – before diving into a detailed presentation of its performance – give a brief outline of the final design.

### 3.1 General design considerations

The general FSP workflow – in both the conventional and the automated processes – consists of the steps depicted in Fig. 1. These were integrated into a consolidated platform, which will be described in detail in the following sections.

The design of the setup was heavily influenced by knowledge gained from previous generations of FSP reactors in our laboratory, but also guided by the requirements derived from its current and future use cases. Standard design guidelines^39^ were followed to define the needs and derive specifications as detailed in the SI.

First and foremost, AutoFSP must be safe to operate under any set of conditions and return to a secure state in case of any emergency or process deviation. Furthermore, accuracy and batch-to-batch reproducibility, as well as the elimination of any carryover between batches are essential prerequisites to make the setup and its automation worthwhile. Finally, any material used for the construction of AutoFSP needs to be chemically and thermally compatible with its contacting media. This is especially important when using tetrahydrofuran in the precursor mixtures as most common polymers are incompatible with this strong solvent, or when considering the high temperatures that occur inside the reactor setup during pyrolysis. A broader overview of the considerations made in the design process is presented in the SI.

**Fig. 3:**
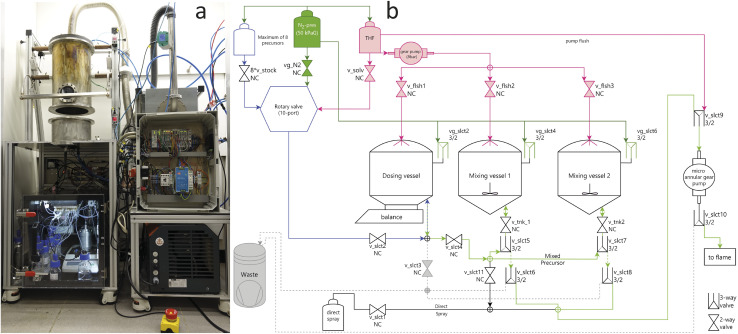
(a) The AutoFSP setup is composed of the reactor (top left), the pneumatic dosing & mixing unit (bottom left), the vacuum pump required for particle collection (bottom right), the enclosed PLC housing (middle right) and the HEPA filter with an attached air flowmeter (top right). (b) Piping and instrumentation diagram (P&ID) of the dosing & mixing unit. Lines in blue and light green are reserved for pure and mixed precursors, respectively. Dark green lines and valves are used for vessel N_2_-pressurization. Grey-dashed are waste lines. Pink lines are used for provision of pure THF for vessel cleaning.

A photographic overview of AutoFSP is given in Fig. 3a. The setup can be considered as merger of two units: an enclosed reactor and the mixing unit, which provides the compounded precursor mixtures for pyrolysis. The interplay of these two units is concerted by an industry-standard programmable-logic-controller (PLC), ensuring process stability, and paying tribute to the inherent safety requirements set by operating an open flame in a laboratory setting. In the following sections, we will present the selected hardware and the workflow performed thereon.

### 3.2 Mechanical design

#### 3.2.1 Layout of the mixing unit

The mixing unit is designed as a pneumatically operated hydraulic system. Liquid movement is achieved by pressurizing the source vessel with N_2_-gas at +500 mbar while maintaining the destination vessel at atmospheric pressure. A useful side effect of using N_2_ instead of air is the mitigation of any flammability risks from highly volatile and flammable THF.^40^ Initially, annular-gear or peristaltic squeeze pumps were considered viable options for liquid movement, but provided no significant advantage, yet several disadvantages (*e.g.*, inferior rinsability and higher cost) compared to the present system, whose layout is depicted as a Piping and Instrumentation diagram (P&ID) in Fig. 3b.

An important design feature of the system is task parallelization. For example, a mixture is compounded while the system simultaneously performs pyrolysis of the preceding batch. The compounding step should be as fast as possible, while preventing any carryover between batches. To this end, a three-vessel design was selected: one tank sitting on an analytical balance is used to gravimetrically compound the mixtures, and two more tanks on magnetic stirring plates are used to mix the precursors thoroughly. They act as buffer tanks to hold the mixture ready prior to pyrolysis. With this design, the dosing step is decoupled from the pyrolysis step, and the total time needed for a production campaign is significantly reduced.

The corrosiveness and dissolving power of the solvents used in the precursors require the use of fluoropolymers (FFKM and FEP) in all wetted parts to ensure long-term performance. This not only restricts the selection of valves but also impacts the design of the vessels, which will be explained in detail in the next section.

#### 3.2.2 Considerations for the design of dosing & mixing vessels

**Fig. 4:**
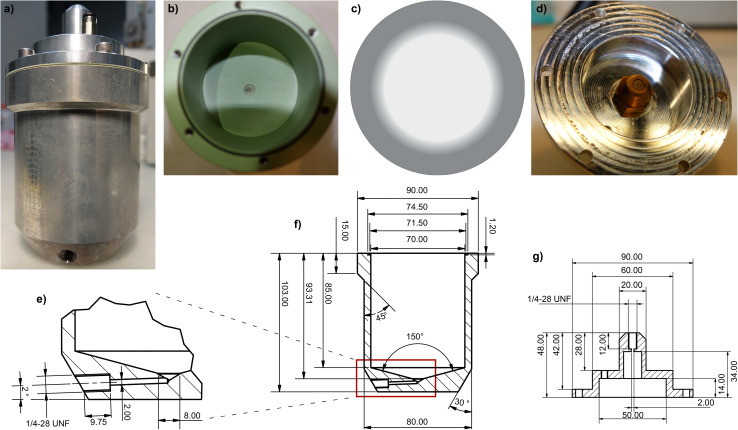
(a) Dosing/mixing vessel made from aluminum. (b) The inside surface is coated with PTFE. (c and d) The cleaning nozzle is installed in the vessel lid and produces a hollow spray pattern. (e) The coned vessel bottom and the sloped bore guarantee complete purging of vessel contents. (f) CAD-drawing of the vessels. (g) The lid hermetically seals between −1 and +2 bar and is held in place by six M6 bolts. All units are in mm, and connection of PTFE tubing is achieved with 1/4-28 UNF fine threads.

The pneumatic handling of liquids requires a vessel design that allows filling, emptying, thorough mixing, and cleaning without disassembly. Therefore, a cup design with a cone-shaped bottom, as shown in Fig. 4, providing a maximum usable volume of 150 mL was considered suitable for the expected batch size. In this layout, liquid precursors flow into and discharge to completeness through an angled duct tapped into the bottom shoulders of the vessel (Fig. 4e). The vessel lid (Fig. 4d and g) is machined from the same material and is secured in place by six M6 bolts. The vessel can be pressurized with N_2_ gas through a push-in fitting in the lid. Gas-tightness is achieved up to an internal pressure of +1 bar using a standard 70 × 3 mm O-ring. For cleaning purposes, a nozzle providing a fine spray of THF is built into the lid.

From a selection of materials, aluminum was considered optimal for machining the vessels because it has excellent mechanical properties at a favorable cost. A detailed list of the other materials considered for this use can be found in the SI. To override the tendency of aluminum to oxidize under corrosive conditions, all inner surfaces were coated with a 25 μm layer of PTFE (Buser Oberflächentechnik AG, Wiler, Switzerland). The coating improves vessel purging by lowering surface wettability and friction on stir bars. Worn coatings are easily reconditioned, with many suppliers offering this service. The lid and top nozzle lack a PTFE coating since they do not come into contact with corrosive precursors. All three vessels are identical, except the dosing vessel, which does not contain a stir bar.

#### 3.2.3 Cleaning procedures of the dosing & mixing vessels

To enable seamless execution of the automated production workflow, a dependable cleaning protocol was established. Therein, the vessel contents are purged, its walls are flushed down with 1 mL s^−1^ of nebulized THF from the top-installed nozzles (Fig. 4d), followed by another purging step. During THF injection and purging, the vessel internal pressure is held at +500 mbar to reduce volatilization of the fine solvent spray. A wide spraying angle of 80° and the choice of a nozzle with a hollow spray pattern (Fig. 4c) ensure effective removal of any leftover precursor, as shown by the quantification of batch-to-batch contamination in the Results and discussion section. The cleaning cycle is repeated three times and takes approximately 30 seconds per cycle.

#### 3.2.4 Considerations for the reactor design

The reactor is shown in Fig. 3a. It consists of a water-cooled lid holding the filter for product recovery, the reactor housing with a sight glass, and the bottom housing connected to the air intake. The flanged parts can be lifted individually using a lever arm construction, enabling access for cleaning, inspection, and filter changes. Under operating conditions, the reactor remains closed to reduce particulate and noise emissions. It is airtight at ambient pressure as the elements compress the O-rings installed within the top and bottom flanges. To remove any foreign dust particles, the inlet air (6–45 m^3^ h^−1^) is HEPA filtered before entering the reactor. The minimum allowable airflow is restricted to mitigate particle recirculation through the flame and to reduce heat accumulation inside the reactor.

Liquid precursors are delivered to the flame with a calibrated micro-annular gear pump, ensuring accurate, repeatable flow rates. The precursor-to-dispersion oxygen flow ratio is key for controlling the final particle size.

#### 3.2.5 Bill of materials for building the AutoFSP setup

The bill of materials can be found in the SI and more details on software and hardware of AutoFS are available in the Experimental section. Most components are sourced from industrial applications, ensuring greater availability and improved cost efficiency.

### 3.3 Automation of tasks

#### 3.3.1 Order workflow organization and task execution

The processing of a batch by AutoFSP can be thought of as a stage-gate-process where each batch must pass a series of process steps and critical decision points on its way from being a digital array of molar fractions to a physical one. Intrinsically, a batch can only be subject to one operation at a time, and the sequence in which tasks are executed is strictly defined (*e.g.* pyrolysis can by definition only be performed after a batch has been compounded). Yet, the setup may perform certain operations on separate batches in parallel to reduce the overall process time.

Therefore, on a source-code level, the tasks were structured in four separate POUs (program organizational units^41^): data management, dosing and mixing, reactor operation, and main program. These can be thought of as independent programs, each with individual variables and subtasks. For example, the POU “Reactor operation” hosts subtasks like “Filter change” and “Flame ignition”, whereas the POU “Data management” includes a read-and-write function for data files. A detailed description of the program structure and the POU subtasks can be found in the SI.

**Fig. 5:**
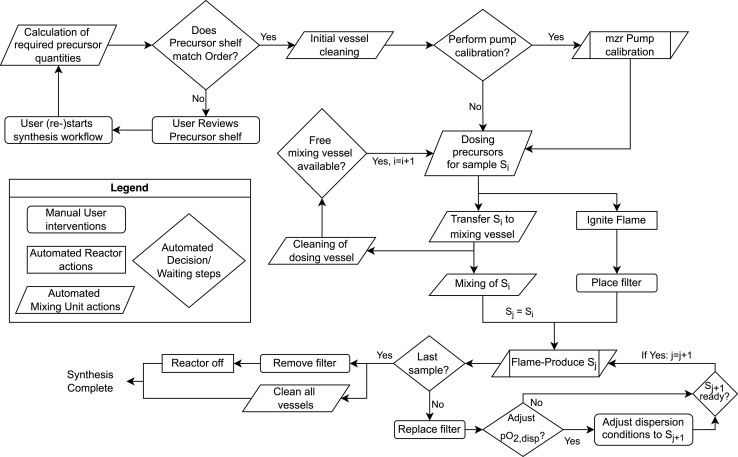
Workflow of batch preparation. Compounding and flame-synthesis are executed in parallel if a free buffer vessel is available. Otherwise, dosing operations remain on hold until a buffer tank is freed up. All automated decision steps incur practically no waiting time unless there are hardware or process limitations. As soon as the condition to proceed is fulfilled, the hold is ended, and the subsequent process step is performed without delay. For safety reasons, the manual user interventions require settlement by the operator within the given safety interval of 90 seconds. If no timely reaction occurs, the system automatically returns to a safe state by ending the pyrolysis, shutting off all gas supplies, and thus putting out the flame.

The structuring in individual POUs allows for a clear separation of the respective processes while decreasing overall process time by parallelizing operations. The particular steps performed during a production campaign are depicted in Fig. 5 and will be explained in detail in the next subsections.

#### 3.3.2 Preliminary calculations

The workflow processing starts with the user uploading a standardized .csv file containing specifications for the materials to be made, limited to a maximum of 14 batches per run. This is called the “Order”. It is matched with a second .csv file, called “Precursor shelf”, containing information about the physically connected precursor bottles. The system verifies the match and checks compliance with hardware-given limitations such as maximum batch size or minimum allowable dosing quantities. If multiple precursors of the same element are available at different concentrations, the precursor that results in dosing of the “optimal” amount is selected. If precursor shelf and order mismatch, a prompt is issued, asking the user to adjust their selection by either providing a different set of precursors or reviewing the order.

#### 3.3.3 Order execution

Once processing of an order has started, all three vessels are subjected to an initial cleaning procedure, and the micro-annular gear pump is automatically calibrated.

The production of a batch begins with gravimetric dosing of up to four pre-provided liquid precursors in specified molar ratios. All weights and related molar metal loadings are logged in a standardized file for reliable documentation, free from errors.

After dosing, the mixture is transferred into a free mixing vessel serving as a buffer and blending vessel. To enable this step, compounding of a new mixture only starts if a free mixing vessel is available. After liquid transfer is complete, the mixture is stirred with a PTFE-coated magnetic stir bar that stays inside the vessel. Meanwhile, the dosing vessel is automatically rinsed and prepared for the next batch.

During stirring of the mixture, the operator is prompted to confirm the ignition of the flame in the reactor. Human intervention is only necessary for safety reasons and underscores the regulatory challenges faced in creating fully autonomous laboratories.

Using a piezo spark igniter, the support flame is ignited, followed by manual adjustment of O_2_-dispersion pressure, and placing the glass-fiber filter for product recovery. Once nanoparticle production from the designated buffer tank has started, process parameters such as temperature, dispersion and filter differential pressures, air flow rate, and O_2_ and CH_4_ flows are continuously logged in a standardized file. If critical reaction parameters deviate beyond permitted ranges, the respective process is paused, and the operator is prompted to address the issue within a given time. Such parameters include for example abrupt temperature changes within the reactor (*e.g.* in case of a plugged injector needle), changes in *p*_O_2_,disp_ due to accumulation of debris in the nozzle or an exceedingly high Δ*p*_filter_ as the filter fills up. If the operator does not correct the situation within the specified time limits, AutoFSP will automatically end all hazardous processes and return to a safe state.

Beyond such deviations, all processes run automatically and require no user intervention except for filter changes at the end of each batch's pyrolysis. These involve removing the current filter, placing a new one, and confirming the correct placement through a prompt. While the subsequent batch is being pyrolyzed, the operator has time to recover the powder product from the filter by careful scraping. Once compounding and pyrolysis of all batches are completed, the reactor is automatically shut down after removal of the last product filter, and all vessels undergo a final cleaning sequence.

### 3.4 Addressing repeatability: AutoFSP demonstration and build verification

Four production campaigns, as shown in Fig. 2 in the Experimental section, were run to quantify robot accuracy and rationalize the extent of unwanted batch-to-batch carryover. The campaigns covered a range of nominal In or Zn loadings over two orders of magnitude.

The resulting products (In_*x*_Zr_1−*x*_O_*y*_ and Zn_*x*_Zr_1−*x*_O_*y*_) were subjected to compositional analysis *via* ICP-OES and determination of specific surface area *via* BET. XRD patterns were used to calculate average crystallite size and analyze the similarity of the patterns resulting from batches of the same nominal composition. In the writing of this manuscript, terms referring to accuracy, trueness, and precision were used as defined in ISO 5725-1 and ISO 5725-2.

## 4. Results and discussion

### 4.1 Accuracy and batch-to-batch repeatability of AutoFSP

#### 4.1.1 Compositional analysis *via* ICP-OES

**Fig. 6:**
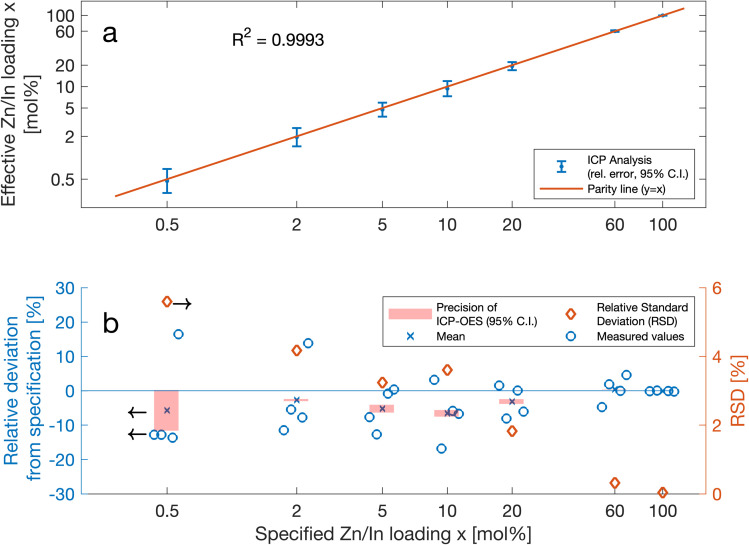
(a) Each blue dot depicts the mean effective loading derived from the four distinct synthesis batches with the same nominal specification (two from the Zn_*x*_Zr_1−*x*_O_*y*_ group and another two from the In_*x*_Zr_1−*x*_O_*y*_ group). The error bars represent the respective 95% confidence interval. The coefficient of correlation between all measured loadings and their respective specifications is *R*^2^ = 0.9993. The closer a data point falls to the parity line, the better it matches its specification. Accordingly, the investigated batches closely correspond to the specified composition, underscoring the accuracy of AutoFSP in matching the specification. (b) Each blue dot represents the relative deviation of the effective Zn/In loading of a given batch from its specification. The uncertainty of the measurement arising from the variability of the analytical method (ICP-OES after microwave digestion) is depicted as light red intervals (95% C.I.) around the mean. The latter is a good indication of the maximum precision that can be reached with the analytical method under given conditions. The diamond-shaped markers represent the relative standard deviation of the average batch composition. It is a good indicator of the precision of the synthesis. Altogether, these indicators suggest that AutoFSP reliably matches the specified product composition over a range of two orders of magnitude (0.5–60 wt%) with a reproducibly high accuracy and precision.

The materials retrieved from the four production campaigns defined in Fig. 2 were used to determine how accurately AutoFSP can match the composition of its effective output with the specifications set in the input file. Therefore, for each nominal loading *x*, a total of four batches were produced as defined in Fig. 2. Their effective compositions were determined *via* ICP-OES, and the corresponding 95% confidence interval was derived (*n* = 4). These results are depicted in Fig. 6a. The individual data points fall onto or very close to the parity line and thus suggest that effective and specified nominal loadings strongly correlate. This observation is further corroborated by a correlation coefficient only marginally different from 1 (*R*^2^ = 0.9993).

To gain a closer insight into the accuracy of AutoFSP, we analyzed the dispersion characteristics of the products generated. The effective loadings for all individual batches are depicted in Fig. 6b. The bias of effective loadings as a metric for the trueness is within ±5% relative to the respective specification across the entire range of loadings. This suggests that AutoFSP produces very little systematic error and is in line or exceeds most other routes commonly used to prepare mixed metal oxide catalysts.^42,43^ The RSD as a measure for the dispersion of the individual effective batch compositions around their mean varies between 5.5% at a nominal loading of 0.5 mol% and less than 1% at a nominal loading of 60 mol%, suggesting a high overall output repeatability, which again is in line or exceeds other routes used for catalyst synthesis.^42,43^

To create a metric for the magnitude of the error introduced by the analytical method itself, each individual batch was analyzed in triplicate. This allowed for the establishment of a 95% confidence interval for the precision of ICP analysis at each nominal loading, depicted as light red intervals in Fig. 6b. As expected, these intervals narrow down as nominal loadings increase, and become insignificant at nominal loadings equal to or above 2 mol%. The relatively large interval and thus high uncertainty observed in the quantification of the 0.5 mol% nominal loading can be explained by the limited solubility of ZrO_2_ in the acid mixture used in microwave digestion. This causes the concentration of Zn or In in the analyte to be close to the LOQ.

Overall accuracy in terms of the overall average relative deviation from the specified loadings, thus considering the entire range of nominal loadings, is 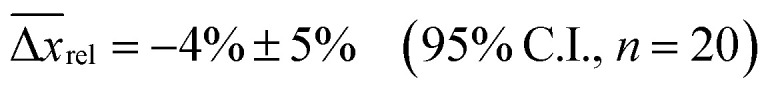. The origin of this slight bias is most likely explained by the limited accuracy of the determination of metal loadings in the precursors (In: ± 0.8%, Zn: ±0.5%, Zr: ±2.3% relative error, each as 95% C.I., *n* = 3), by the limited accuracy of the balance readings – especially under dynamic weighing conditions – and by the algorithm active during dosing of precursors. Specifically, to accelerate the dosing process for a batch, the addition of a precursor is halted if less than 0.1 g of it is missing compared to the pre-calculated amount. To reduce the impact of such deviations during dosing, the minimum allowed dosing quantity per precursor is set at 3.0 g, which corresponds to a maximum theoretical “underdosing” of 3.3%. Optimizing this hard-coded endpoint could further improve the accuracy of AutoFSP.

Moreover, replacing the current balance model by a more suited type, tailored for dynamic weighing could make a step in the same direction. Although the certified accuracy of the balance is ±10 mg under ideal, static conditions, it can be expected that the reading is much less exact under dynamic weighing conditions.

Yet, these findings are relativized by the fact that AutoFSP can – unlike any other comparable method – provide user-specified materials, composed of up to four elements over two orders of magnitude, and the precision reached on each element falls in the same order of magnitude, regardless of its chemical characteristics.

#### 4.1.2 Repeatability of specific surface area

**Fig. 7:**
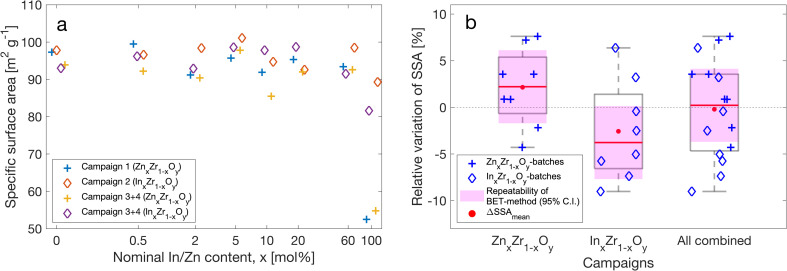
(a) Individual BET-surfaces for all 28 batches issued from the four production campaigns represented by four different marker types. (b) Box plot of the relative batch-to-batch variation of specific surface area (SSA) for pairs with the same composition issued from separate production campaigns.

Specific surface area (SSA) is not only relevant for tweaking the reactivity of materials and maximizing the use of active metals in catalysts – it also reveals information about the material's synthesis and particle size. Therefore, we carried out systematic SSA measurements of the materials obtained from the four production campaigns shown in Fig. 2, and present the results in Fig. 7a.

Since the SSA depends quite strongly on the composition of a material, with all other production parameters unchanged, a batch-to-batch (B2B) comparison of each pair of two materials with the same compositions was performed. For example, we would relate pairs of SSAs of the two In_5_Zr_95_O_*y*_ batches retrieved from campaign 2 (C2-S3) and campaign 4 (C4-S2), and so on.

The relative B2B differences for all pairs are shown in Fig. 7b, categorized by their elemental composition. A B2B repeatability relative standard deviation of AutoFSP, RSD_AutoFSP,BET_ = 4.5% was derived, which again confirms a relatively good repeatability of the setup's output in terms of surface properties. Systematic campaign-to-campaign precision seems to be undermined by minute deviations in the process conditions during the pyrolysis step which seem to introduce a systematic bias to all batches of that campaign.

Furthermore, BET as an analytical technique may also contribute in part to the random and systematic variations observed. Therefore, we examined the precision that could be reached on the BET-equipment available in our laboratory by performing four repetitions on four different batches of pure ZrO_2_ (including sample preparation, degassing, and BET-analysis). The average repeatability standard deviation – *S̄*_r_ – was derived and translated into the benchmark repeatability relative standard deviation, RSD_BET_ = 3.6%. This was converted into the corresponding 95% confidence interval, as depicted in Fig. 7b. While this finding is in accordance with the published range of 0.10–4% (ref. 44) it also confirms that the limited precision of the analytical technique may contribute significantly to the variation observed in the abovementioned B2B comparison.

In summary, AutoFSP can be used to produce materials with surface properties as repeatable as the measurement technique itself, which in turn demonstrates its excellent performance in terms of the repeatability of syntheses performed thereon.

#### 4.1.3 Crystallite size by XRD

**Fig. 8:**
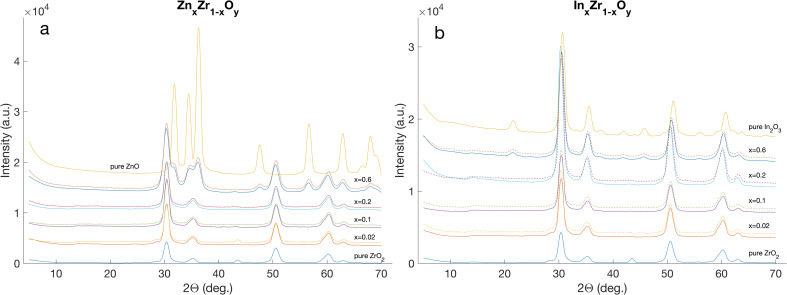
Stacked pairs of XRD patterns of all materials retrieved from the four production campaigns of Zn_*x*_Zr_1−*x*_O_*y*_ (a) and In_*x*_Zr_1−*x*_O_*y*_ (b). The dashed lines represent the corresponding replication batches with the same nominal composition.

The XRD-pattern of a compound not only includes phase information, but also details about the mean crystallite size, and to a certain extent the “heat history” a particle has seen during its flame synthesis and subsequent rapid cooling. Therefore, by comparing two patterns, the similarity of two materials and potentially the similarity of the preceding process during its synthesis can be assessed. All pairs are shown in Fig. 8.

The similarity observed in all XRD patterns of Zn_*x*_Zr_1−*x*_O_*y*_ for *x* ≤ 0.2 suggests that Zn and Zr are homogeneously mixed and no distinct ZnO phase occurs in the material. The dominant phase seems to be tetragonal ZrO_2_. The same seems to hold true for In_*x*_Zr_1−*x*_O_*y*_ when *x* ≤ 0.6. Once Zn becomes the dominant species, the crystallography changes from a cubic to a wurtzite type.^45^ In contrast to this, In_2_O_3_ crystallizes in a cubic crystal structure which produces a pattern very similar to that of pure ZrO_2_.^46^

**Fig. 9:**
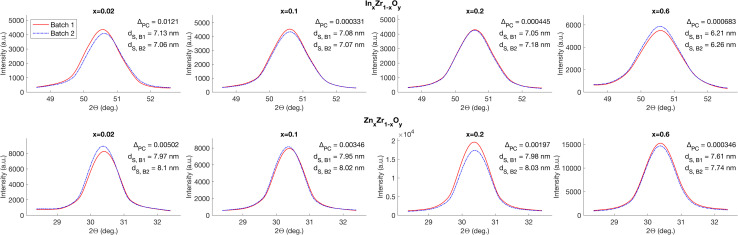
Comparison of characteristic XRD peaks for batch pairs with identical nominal loadings issued from separate production campaigns. The peak selected for materials of types In_*x*_Zr_1−*x*_O_*y*_ and Zn_*x*_Zr_1−*x*_O_*y*_ is at 2*Θ* = 30.4° and 2*Θ* = 50.5°, respectively. As a measure of similarity, the Procrustes distance between the two overlaid lines was calculated for each pair, denoted as *Δ*_PC_. Furthermore, the mean crystallite size as calculated by using the Scherrer equation is denoted as *d*_s_. The data depicted above are unedited and directly extracted from the raw measurements. Note that line broadening may also be caused by strain, but this cause of line broadening is not considered here.

For both In and Zn, a peak in the respective group of XRD patterns was selected, such that it had no overlay with any other peaks and the peak shoulders could be clearly distinguished (peaks at 2*Θ* = 30.4° for In_*x*_Zr_1−*x*_O_*y*_ and at 2*Θ* = 50.5° for Zn_*x*_Zr_1−*x*_O_*y*_). The patterns in the region around these 2*Θ* were overlaid for visual shape and size comparison as detailed in Fig. 9. Qualitatively, a very high similarity between pairs can be observed. Furthermore, with the help of Procrustes analysis, a relative difference, *Δ*_PC_, was established for each pair. The results of the analysis are presented in the respective subplots and for all pairs, a Procrustes difference *Δ*_PC_ < 0.005 holds true, suggesting a very high similarity of shape pairs.

Moreover, the crystallite size (denoted as *d*_s_ in Fig. 9) for the same selection of materials was calculated by means of the Scherrer equation using the full width at half maximum (FWHM) of the peaks around the above-mentioned selected 2*Θ*. The standard deviations of the mean crystallite size of each pair of patterns were combined into a common average Relative Standard Deviation RSD_CS_ = 0.8% which suggests a very high repeatability of the crystalline properties.

The high similarity of the XRD patterns within the pairs corroborates the assumption that flame conditions during operation of AutoFSP are highly repeatable and that the “thermal trajectory” a particle sees is equally repeatable.

### 4.2 Batch-to-batch carryover

Unintended carryover between batches can be a serious deviation and substantially affect the catalytic activity or other properties of the product. Since the hardware of AutoFSP repeatedly contacts all batches during a campaign, this risk is genuine and should not be overlooked. Consequently, the design of both software and hardware is aimed at reducing carryover to an acceptable minimum, where an impact on the product properties can be ruled out.

To this end, four batches listed in Table 1—each from different production campaigns—were examined for contamination by elements used in the previous batch. The observed analyte concentrations were so close to the LOQ of the respective elements that their significance must be interpreted with caution. The overall carryover amounts to around 0.1 mol% of the following batch, with the ratios of the contaminants matching their ratio in the previous batch (for example, a production of In_0.2_Zr_0.8_O_*y*_ results in a carryover of 0.07 mol% Zr and 0.016 mol% In to the next batch). Overall, the observed carryover was in the same order of magnitude as the purity of the used precursors. If higher purities are required, this can most likely be accomplished with the use of high-purity precursors and by increasing the number and duration of flush cycles between batches with different compositions.

**Table 1:** Quantification of carryover *via* ICP-OES

Batch ID	Composition	Preceded by:	Contamination found [mol% relative to the main phase]		
			Zn	Zr	In
C1-S7	ZnO (100%)	Zn_0.6_Zr_0.4_O_*y*_		0.11	
C2-S8	ZrO_2_ (100%)	In_2_O_3_ (100%)			0.07
C3-S7	ZnO (100%)	In_0.2_Zr_0.8_O_*y*_		0.070	0.0163
C4-S3	In_2_O_3_ (100%)	In_0.05_Zr_0.95_O_*y*_	0.016	0.122	

### 4.3 Production speed and efficiency

**Fig. 10:**
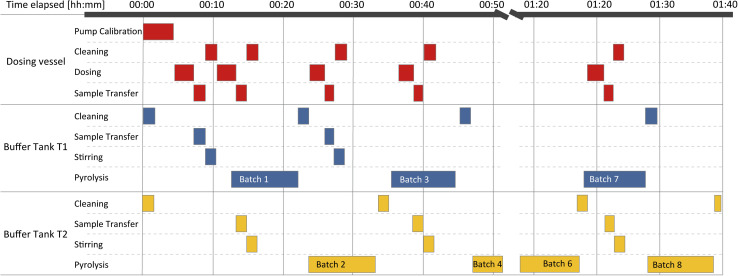
Gantt chart of process steps during production campaign 4. The rate limitation of the overall production speed by the pyrolysis step is worth noting, which in turn cannot be shortened further, but is a given parameter. Shown here are the preparation of precursor mixtures and the pyrolysis of batches 1, 2, 3, and 8.

The efficiency gain from switching from traditional methods to AutoFSP is significant. The units of operator work time required per campaign are typically cut by a factor of two to three. The main reductions come from decreasing the overall attendance time and automating initial calculations and all documentation tasks. The estimated process times during Campaign 1 are shown in a Gantt chart in Fig. 10.

In the current design, AutoFSP operates at nearly maximum speed, with pyrolysis being the rate-limiting step. Dosing, transfer, and stirring of the next batch are faster than pyrolysis, so they don't affect overall process times. The processing of Campaign 1 took about 100 minutes, with an additional 50 minutes of preparative tasks, totaling approximately 150 minutes of operator attendance. During AutoFSP operation, the operator changes the filters and manually collects the powder from them, making the process straightforward and easy to manage.

These tasks typically require only 2–3 minutes per batch, leaving around 6 minutes per batch for the operator to perform other tasks in the vicinity of the reactor. In contrast, the conventional process demands full operator attendance for supervising parameters, reconnecting bottles, and documentation. Attendance would total about 5 hours without allowing the operator to perform any tasks in parallel. The effective durations of the real-life syntheses were clocked and are presented in the SI.

Since all preliminary calculations and process documentation are automated, human errors are prevented, averting any lengthy post-synthesis correction in case of deviations.

#### 4.3.1 Efficacy *vs.* cost

The bill of materials, along with its associated costs, is available in the SI. The construction of the “base” setup (*i.e.*, without automating any process steps) required an expenditure of roughly 41 000 CHF. Implementing AutoFSP incurred an additional cost of 14 000 CHF. Considering that AutoFSP enables a more than two-fold acceleration of novel material development, while also improving process stability, ensuring rigorous process documentation, and further freeing time resources, the costs for automation compare very favorably.

Out of the costs incurred for automation, the bulk is associated with the purchase of THF-compatible microfluidic valves with fluoropolymer seals. The use of THF for vessel cleaning and to decrease the viscosity of precursor mixtures is indispensable. To date, no sealing material with similarly good compatibility to this solvent is available on the market. Replacing THF with a suitable alternative could significantly reduce the investment required for setup automation.

#### 4.3.2 Flexibility *vs.* robustness

The current design of AutoFSP allows the setup to be used in either conventional or automatic production. On one hand, the automatic mode was designed to be as efficient as possible and provide repeatable results as previously presented. On the other hand, pyrolysis of a manually prepared precursor mixture is possible without any impact on product properties. This is especially important to produce single-batch campaigns or when precursor stability upon mixing (*e.g.*, unwanted precipitation) is not guaranteed. In the current design, the operator can switch between the two modes without any hardware changes.

#### 4.3.3 Speed and traceability

Traceability is ensured by live recording and logging of all reaction parameters during synthesis. This makes each production step traceable and prevents operator errors in the process documentation. The amount of data generated is more comprehensive as process parameters are logged continuously and saved in a standardized, machine-readable .csv file. This is especially useful for the integration of AutoFSP into a more comprehensive MAP, which requires a very high degree of standardization.

### 4.4 Safety of AutoFSP

AutoFSP's main safety concerns include flammability of the precursors, unwanted formation of flammable air/gas mixtures, rupture of pressurized glass bottles, and user toxicity of nanomaterials. Flammability hazards are largely eliminated by using N_2_ gas for inertization of the mixing unit and 3-layer spatial separation of flammable precursors from the flame (flame inside a closed reactor, precursors inside inerted glass bottles inside a closed mixing unit, and spatial separation of the reactor and mixing unit). The presence of multiple safety layers ensures that the setup can still be restored to a safe state if any of the layers are compromised (*e.g.*, precursor bottle shattering or fuel line catching fire).

For example, hardware was selected in such a way to return to a safe state upon shutdown in case of an emergency. Specifically, all valves and mass flow controllers are shut, the pumps are turned off, and the pressure on the precursor bottles is released.

Risks associated with the pressure induced shattering of the precursor glass bottles (*i.e.*, in case of a failure of the pressure regulator) are mitigated by enclosing the bottles within the housing of the mixing unit, by the use of an overpressure release valve, and by utilizing pressure-proof glass bottles (100–1000 mL, −1 to +1.5 bar, Duran®, Pressure plus+, DWK Life Sciences, Germany).

Nanoparticle toxicity risks are a serious safety concern during production of such materials, especially in a small-scale laboratory setting with non-continuous process operation. The frequent opening of the reactor and fume hood, which is inevitable for filter changes, potentially allows nanoparticles to escape to other parts of the laboratory. The study performed by Demou *et al.* gave valuable insights about the mechanisms of unwanted nanoparticle release and helped to draft the closed reactor design and develop a workflow routine enforced by AutoFSP.^47^

The current effective airflow rate defines the required waiting time before opening the reactor for filter changes. Less airflow means longer wait times are required to remove lingering particulates. Additionally, the vacuum pump exhaust is located near the ventilation intake at the back of the fume hood, allowing direct removal of potentially contaminated off-gas. During operation, contamination of laboratory air is monitored by particulate counting devices, giving out a warning if critical thresholds are exceeded.

## 5. Conclusions and outlook

In this work, we introduce AutoFSP, a platform for the automated flame synthesis of pure and complex MMO nanoparticles. AutoFSP has already proven helpful in optimizing a real-world catalyst system by providing a systematic array of In_*x*_Zr_1−*x*_O_*y*_ and Zn_*x*_Zr_1−*x*_O_*y*_. It could be a powerful and promising tool for faster discovery of new materials.

Looking ahead, AutoFSP could become a key synthetic tool within a materials acceleration platform, advancing towards fully autonomous labs. Current research offers guidance on connecting AutoFSP's physical lab operation with essential data integration and optimization pipelines.^48^

Automation of FSP as in AutoFSP can be considered a crucial step in closing the gap between *in silico* prediction of material properties, such as catalytic performance or surface characteristics, and experimentally driven, physical data collection. For these models to provide accurate predictions, large quantities of physically obtained training data are required. For the experimentalist, an “educated guess” and capable, efficient hardware are preliminary to get the job done. The acceleration of materials provision enabled by AutoFSP can be leveraged for systematic screening of catalyst performance, as in the current study, but also for the discovery of new materials.

Current safety requirements at most research institutions prohibit the unsupervised operation of flames in a laboratory due to the inherent fire hazard. Such policies are unlikely to change in the future. Therefore, complete automation of FSP (including filter change) seems unreasonable or would provide an unfavorable effort-to-benefit ratio. Yet, AutoFSP has paved the way to reliably deliver materials according to specifications. It operates accurately and reproducibly, and at lower overall cost, including labor.

Future developments may focus on incorporating AutoFSP into completely autonomous laboratory workflows. These require a higher degree of automation in predicting material performance using AI-based models, as well as accelerated catalyst testing. Another path of innovation could stem from adapting the design concepts presented in this manuscript to FSP under reducing conditions, thereby providing access to metallic particles.

## Author contributions

K. M. E., P. O. W. and W. J. S. conceived AutoFSP. K. M. E. wrote critical software for its operation and conceived the user interface. K. M. E. designed and performed all experiments for build verification with helpful input from R. N. G. P. O. W. assisted with de-bugging and contributed critical input to improve the design of software and hardware. W. J. S. led the research team and acquired funding. K. M. E. and R. N. G. conducted all data curation and formal analysis. K. M. E. prepared illustrations and wrote the original draft, while all authors contributed to the review and editing of the manuscript.

## Conflicts of interest

There are no conflicts to declare.

## Supplementary Material

DD-004-D5DD00042D-s001

## Data Availability

Data for this article, including experimental data, hardware details, and software for setup operation, are available at Zenodo.org at https://doi.org/10.5281/zenodo.14721618. Supplementary information is available. See DOI: https://doi.org/10.1039/d5dd00042d.
