# Supplementary material for: An automated platform for “on-demand” high-speed catalyst synthesis by flame spray pyrolysis
Source: Digit Discov. 2025 Oct 10;4(12):3478–91. doi: 10.1039/d5dd00042d (PMC12557465; doi:10.1039/d5dd00042d)
Supplement: DD-004-D5DD00042D-s001 [file DD-004-D5DD00042D-s001.pdf]

Supplementary Information (SI) for Digital Discovery.
This journal is © The Royal Society of Chemistry 2025

Supplementary Information:

Automated platform for "on-demand" high-speed catalyst synthesis by Flame Spray Pyrolysis†

Konstantin M. Engel[a], Patrik O. Willi[a], Robert N. Grass[a] and Wendelin J. Stark[a,*]

[a]Functional Materials Laboratory, Institute for Chemical and Bioengineering, ETH Zürich, Vladimir-Prelog-Weg 1/1-5, 8093 Zürich, Switzerland.

[*]Corresponding author: wendelin.stark@chem.ethz.ch

1. Experimental Methods

Preparation of Indium precursor

Indium precursor was made by dissolving elemental Indium (95.4 g, 99.995%, polymet.de) in HNO_3 (384 g, 68%, Sigma-Aldrich) and evaporating the liquid while stirring until only a slurry of $In(NO_3)_3$ remained. A mixture of 100% AcOH/Ac_2O (1:1 w/w, both Sigma-Aldrich) was added dropwise to the hot slurry accompanied by vivid bubbling and formation of NO_x. After addition of 1200 g of the latter mixture, and keeping at 160°C for 1 h, 2-ethylhexanoic acid (292 g, 2-EHA, Acros Organics) was added. The reaction mixture was distilled with a decreasing pressure profile and end pressure of 50 mbar at 160°C to remove any remaining Ac_2O and AcOH and re-dissolve any precipitated $In(OAc)_3$. Upon cooling, the caramel-brown solution was topped up to a total mass of 830 g with a mixture of 2-EHA/tetrahydrofuran (2:1 w/w) as to achieve a final concentration of 1.0 mol kg^{-1}.

Parameters used for ICP-OES

Table S1: Parameters used for microwave-assisted acid digestion prior to ICP-OES.

<table>
<tr><td>Batch Comp.</td><td>Acid Mixture used (wt. %)</td><td>Volume of acid used</td><td>Settings</td><td>mass of sample [mg]</td></tr>
<tr><td>$In_{0.6}Zr_{0.4}O_x$</td><td>23% H_2SO_4 + 7% $HClO_4$ + 70% H_2O</td><td rowspan="3">3 ml</td><td rowspan="3">700 W
60 min</td><td>10-20</td></tr>
<tr><td>ZnO</td><td>30% HNO_3 + 70% H_2O</td><td>30-40</td></tr>
<tr><td>All other</td><td>66% H_2SO_4 + 23% HNO_3 + 11% H_2O</td><td>20-30</td></tr>
</table>

Table S2: Parameters for the sample analysis via ICP-OES.

<table>
<tr><td colspan="2"></td><td>Zn</td><td>Zr</td><td>In</td></tr>
<tr><td colspan="2">Wavelength used [nm]</td><td>213.857</td><td>357.685</td><td>325.608</td></tr>
<tr><td rowspan="2">Calibration Range [ppm]</td><td>Lower</td><td>0.5</td><td>0.5</td><td>5</td></tr>
<tr><td>Upper</td><td>10</td><td>10</td><td>100</td></tr>
<tr><td colspan="2">LOD in analyte [ppm]</td><td>0.13</td><td>1.36</td><td>6.10</td></tr>
<tr><td colspan="2">LOQ in mixed metal oxide [mol%]</td><td>0.08</td><td>in ZnO: 0.16
in In_2O_3: 0.09</td><td>0.37</td></tr>
</table>

2. AutoFSP overview

Table S3: Needs and specifications determined for the design of AutoFSP.

Essential:
• Accuracy and batch-to-batch reproducibility should be within or exceed performance of the conventional process.<br>• The setup must be safe to operate and automatically return to a safe state if any emergency trigger is activated.<br>• Production of multiple batches in a row with varying loadings and dopant types must be accomplished without carry-over of traces from one batch to another.<br>• Building materials must be inert to corrosivity and dissolving power of precursor solvents and heat resistant if they touch hot reaction gases.
Desirable:
• Easy switch between conventional and automatic mode.<br>• Most parts used for construction are standardized or available from multiple commercial suppliers and thus replaced easily and affordably when broken.
Useful:
• Minimum training required for new users.<br>• The entire setup must fit into the fume hood currently available in our laboratory.

Table S4: Overview of POUs and the programs they contain. The names of the POUs are presented as they appear in the software.

Main Program	POU_Ablauf		POU_Mixing_Spraying		POU_LoadAndSave	
Main Program is being permanently executed, in all modes and under all conditions (supervises process parameters like T, P) and ensures communication with the connected periphery	Always On: function is continuously executed	Manual: contains tasks executed during manual operation	Calibration function in manual mode	CheckIn mixing unit at the end of a campaign	Load Orders	Save Orders
	CheckOut reactor before Campaign	CheckIn reactor after Campaign	Prepare Mixing vessels prior to starting a campaign	Prime the precursor lines, purge any air bubbles	Load Precursor Shelf	Save Precursor Shelf
	Ignite Flame	TurnOffFlame	Abort dosing operations	Clean mixing vessel	Load Settings	Save Settings
	Watch Mixing Vessels: checks if a batch is ready for pyrolysis	Spray: Supervises the pyrolysis	Dosing function	Idle: Monitor, if a free buffer tank is available and batches left for production		
	SynAlwaysOn: constantly executes base tasks required during auto operation.	MZR-calibration	Idle: Mixing vessel full, but no buffer tank available	Transfer compounded precursor mixture to T1/T2		
	Reset Manual	Place Filter	Clean T1	Stirrer On T1		
			Clean T2	Stirrer On T2		

Sample_ID	EN1	EN2	EN3	EN4	trgt_molperc1	trgt_molperc2	trgt_molperc3	trgt_molperc4	trgt_prec_conc	tot_prod_wgth	mzr_flow	O2_flow
					%	%	%	%	mmol/kg	g	ml/min	NL/min
BEGIN												
0.5ZnZr	Zn	Zr			0.5	99.5	0	0	400	1.4	5	5
2ZnZr	Zn	Zr			2	98	0	0	400	1.4	5	5
5ZnZr	Zn	Zr			5	95	0	0	400	1.4	5	5
10ZnZr	Zn	Zr			10	90	0	0	400	1.4	5	5
20ZnZr	Zn	Zr			20	80	0	0	400	1.4	5	5
60ZnZr	Zn	Zr			60	40	0	0	400	1.4	5	5
100Zn	Zn				100	0	0	0	400	1.4	5	5
100Zr	Zr				100	0	0	0	400	1.4	5	5
END												

Figure S1: Input file for which contains the specifications for campaign #1. The file format is ".csv" but depicted here as a table for better readability.

BEGIN				
vlv_nmbr	elmnt_name	conc	init_fill_wgh	cnnct_OK
1	Zr	467	840	TRUE
2	Zn	506	340	TRUE
3	Zn	51	130	TRUE
4	Zn	5	100	TRUE
END				

Figure S2: Input file for which contains the specifications for the precursors used in campaign #1. The file format is ".csv" but depicted here as a table for better readability.

JPR_70.csv				
Date:	Wed, 2024-09-11			
Time:	14:56:30			
Start MZR C.	13:15:25			
Compl. MZR	13:18:44			
Slope	Intercept	R2		
0.0004822	1.378014	0.9972762		
P_cal (mbar	500			
AO_Signal	3276	8190	13106	21300
g pumped in	0.6829986	1.238666	1.686333	2.678335
time_flsh_pu	12	USINT	Sec. - duration of flash pump operation during cleaning processes of mixing and dosing vessels.	
flsh_pump_:	80	USINT	In percent of full power.	
set_n_clear	2	USINT	Number of cleaning cycles when preparing vessels for next sample.	
prge_pos_p	500	UINT	mbar - positive pressure setpoint for any liquid transfers. Refers to rel. pressure above atmospheric.	
stirring_time	60	USINT	Sec. - duration of stirrers operation once a mixture has been transferred to T1/T2.	
dose_mrgn	3.5	REAL	grams - once no more than this weight is missing the dosing process is decelerated.	
max_dsng_(	40	USINT	Sec. - maximum allowed wait time to dose one precursor - used to exit while loop during dosing. Will be extended if the system detects effective flow is still positive.	
min_prec_fil	5	USINT	grams - minimum allowed fill level for precursor bottles. if < than the specification would remain at end of dosing, will not start and prompt user to re-fill bottle.	
avg_prec_d	0.91	REAL	gram/ml - average precursor and solvent density. Used to calculate pump times.	
time_piezo_	5	USINT	Sec. - during which the piezo is on.	
ign_delay1_	18	USINT	Sec. - maximum allowed waiting time for MFCs to adjust to preset auto value. If spec not reached within the given time, MFC are turned off.	
ign_delay2_	5	USINT	Sec. - maximum allowed wait time after ignition until the temperature needs to increase. Otherwise CH4 gas feed is turned off.	
max_user_r	90	USINT	Sec. - maximum wait time for user to react upon prompt. if no timely reaction, the process gets aborted and the machine is sent back to a safe state.	
pdiff_max	-0.6	REAL	bar- maximum allowed pressure difference at filter before warning occurs and process is sent to a break.	
pDispO2_SI	1.5	REAL	bar - setpoint for O2 dispersion pressure.	
pDispO2_w	0.2	REAL	bar - maximum allowed [+/-] deviation of pO2disp from setpoint to activate pressure warning.	
stop_delay1	150	USINT	Sec. - maximum idle time to elapse while no spraying is ongoing before the flame gets turned off.	
Precursor shelf				
vlv_nmbr	elmnt_name	conc	init_fill_wght	final_fill_wght
-	-	mmol/kg	g	g
1	Zr	467	840	636
2	Zn	506	340	266
3	Zn	51	130	108
4	Zn	5	100	84

Sample_ID	EN1	EN2	EN3	EN4	trgt_mass1	trgt_mass2	trgt_mass3	trgt_mass4	trgt_mass_s	prec_conc_	prec_conc_	prec_conc_	prec_conc_	act_mass1	act_r
0.5ZnZr	Zn	Zr			15.36	32.74	0	0	0	5	467	0	0	15.28	
2ZnZr	Zn	Zr			6.05	32.39	0	0	0	51	467	0	0	6.1	
5ZnZr	Zn	Zr			15.26	31.66	0	0	0	51	467	0	0	15.2	
10ZnZr	Zn	Zr			3.12	30.43	0	0	6	506	467	0	0	3.11	
20ZnZr	Zn	Zr			6.43	27.87	0	0	7	506	467	0	0	6.39	
60ZnZr	Zn	Zr			21.92	15.83	0	0	9	506	467	0	0	22.02	
100Zn	Zn				42.31	0	0	0	12	506	0	0	0	42.42	
100Zr	Zr				32.86	0	0	0	6	467	0	0	0	32.78	

Figure S3: Exportable file Format in .csv-format for the completed campaign#1 (left half of .csv). For the other half of the table, see Figure S4.

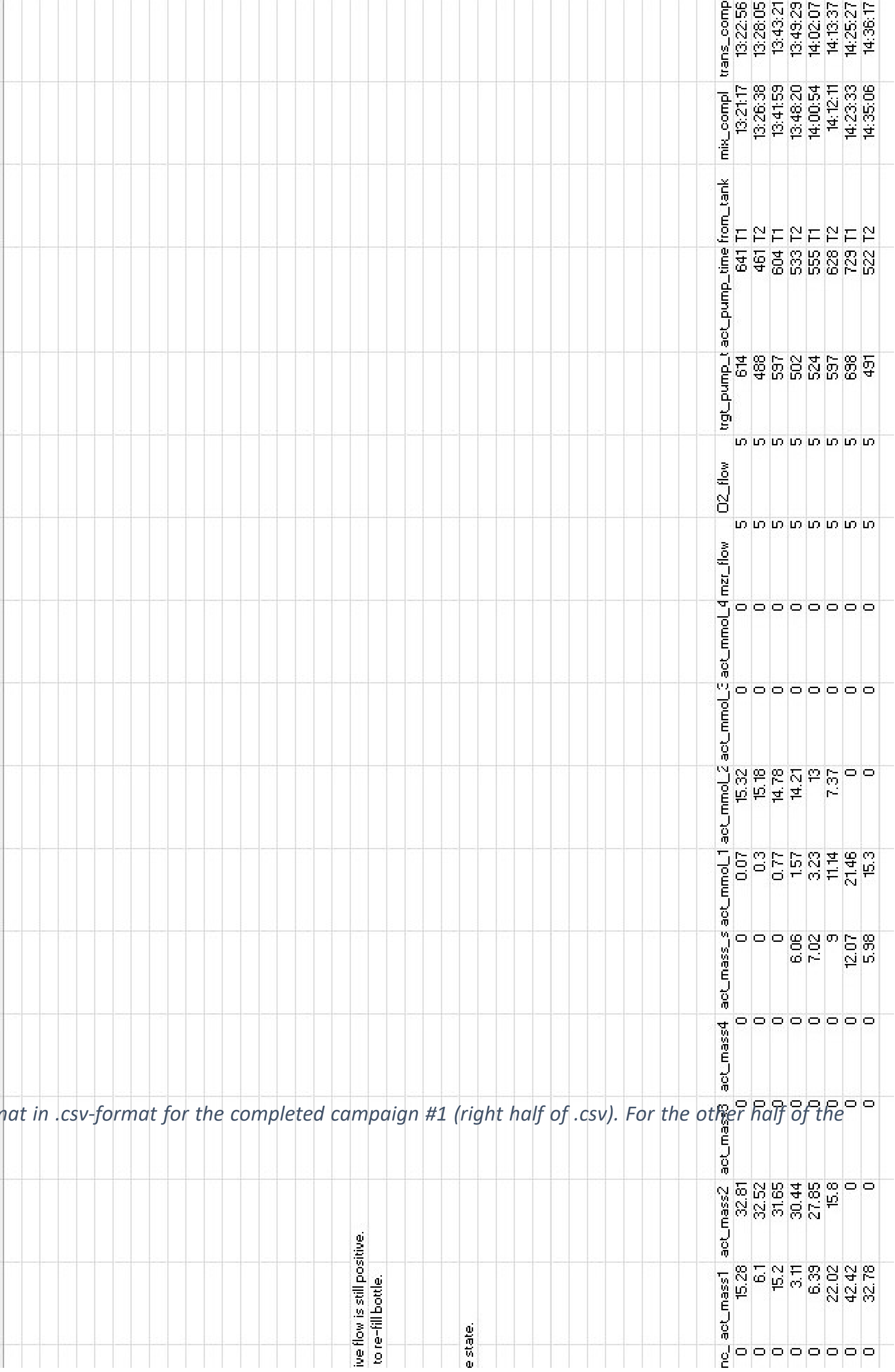

nc_	act_mass1	act_mass2	act_mass3	act_mass4	act_mass_s	act_mmol_1	act_mmol_2	act_mmol_3	act_mmol_4	mzr_flow	O2_flow	trgt_pump_t	act_pump_time	from_tank	mix_compl	trans_comp
0	15.28	32.81	0	0	0	0.07	15.32	0	0	5	5	614	641	T1	13:21:17	13:22:56
0	6.1	32.52	0	0	0	0.3	15.18	0	0	5	5	488	461	T2	13:26:38	13:28:05
0	15.2	31.65	0	0	0	0.77	14.78	0	0	5	5	597	604	T1	13:41:59	13:43:21
0	3.11	30.44	0	0	6.06	1.57	14.21	0	0	5	5	502	533	T2	13:48:20	13:49:29
0	6.39	27.85	0	0	7.02	3.23	13	0	0	5	5	524	555	T1	14:00:54	14:02:07
0	22.02	15.8	0	0	9	11.14	7.37	0	0	5	5	597	628	T2	14:12:11	14:13:37
0	42.42	0	0	0	12.07	21.46	0	0	0	5	5	698	729	T1	14:23:33	14:25:27
0	32.78	0	0	0	5.98	15.3	0	0	0	5	5	491	522	T2	14:35:06	14:36:17

ive flow is still positive.
to re-fill bottle.
e state.

Figure S4: Exportable file Format in .csv-format for the completed campaign #1 (right half of .csv). For the other half of the table, see Figure S3.

	A	B	C	D	E	F	G	H	I	J	K
1	JPR_73										
2	5ZnZr_SynData										
3	24.09.2024										
4	Time	T1 (inlet pump	T3 (outlet reac	T4 (inside reac	pO2disp	dpFltr	Airflow	Flow_O2_disp	mzr pump ml	Mode	Status
5	[-]	deg. C	deg. C	deg. C	barg	barg	Nm^3	NL/min	mL/min	[-]	[-]
6	18:22:15	23	62	68	1.53	0	42.8	4.99	12	Automatic	
7	18:22:20	23	54	62	1.53	0	43	4.99	5	Automatic	OK
8	18:22:24	23	52	58	1.51	0	43	4.99	5	Automatic	OK
9	18:22:29	23	40	59	1.48	-0.05	43	4.99	5	Automatic	OK
10	18:22:33	24	70	80	1.45	-0.06	35.7	5	5	Automatic	OK
11	18:22:38	24	83	103	1.48	-0.06	35.7	4.99	5	Automatic	OK
12	18:22:42	24	88	118	1.48	-0.06	35.5	4.99	5	Automatic	OK
13	18:22:47	25	89	131	1.47	-0.08	35	4.99	5	Automatic	OK
14	18:22:51	25	86	132	1.46	-0.08	35.3	4.99	5	Automatic	OK
15	18:22:56	26	90	138	1.48	-0.06	33.6	5	5	Automatic	OK
16	18:23:00	26	83	140	1.46	-0.08	38.5	5	5	Automatic	OK
17	18:23:05	27	93	146	1.45	-0.08	31.9	4.99	5	Automatic	OK
18	18:23:09	27	95	150	1.45	-0.08	33.8	4.99	5	Automatic	OK
19	18:23:14	28	95	152	1.45	-0.08	32.9	4.99	5	Automatic	OK
20	18:23:18	28	95	153	1.46	-0.1	32.5	4.99	5	Automatic	OK
21	18:23:23	29	95	156	1.46	-0.1	31.7	5	5	Automatic	OK
22	18:23:27	29	95	159	1.61	-0.11	31.3	5	0	Automatic	pDisp O2 warn
23	18:23:32	30	78	141	1.63	-0.08	30.6	5	0	Automatic	pDisp O2 warn
24	18:23:36	30	58	118	1.55	-0.08	30.8	4.99	0	Automatic	pDisp O2 warn
25	18:23:41	31	54	104	1.55	-0.08	31.9	4.99	0	Automatic	pDisp O2 warn
26	18:23:45	32	52	91	1.55	-0.08	31.8	4.99	0	Automatic	pDisp O2 warn
27	18:23:50	32	51	84	1.55	-0.08	32	4.99	0	Automatic	pDisp O2 warn
28	18:23:54	33	51	78	1.56	-0.08	32	4.99	0	Automatic	pDisp O2 warn
29	18:23:59	34	50	74	1.51	-0.08	31.4	5	0	Automatic	pDisp O2 warn
30	18:24:03	34	50	70	1.53	-0.08	31.5	4.99	0	Automatic	pDisp O2 warn
31	18:24:07	35	49	68	1.53	-0.08	31.7	4.99	0	Automatic	pDisp O2 warn
32	18:24:12	36	49	67	1.55	-0.08	31.6	4.99	0	Automatic	pDisp O2 warn
33	18:24:16	36	49	66	1.55	-0.08	31.6	4.99	0	Automatic	pDisp O2 warn
34	18:24:21	37	49	65	1.55	-0.08	31.2	4.99	0	Automatic	pDisp O2 warn
35	18:24:25	37	49	64	1.55	-0.08	31.2	5	0	Automatic	pDisp O2 warn
36	18:24:30	37	49	64	1.55	-0.08	31.3	5	0	Automatic	pDisp O2 warn
37	18:24:34	37	48	63	1.55	-0.08	31.2	4.99	0	Automatic	pDisp O2 warn
38	18:24:39	38	48	63	1.55	-0.08	31.2	4.99	12	Automatic	pDisp O2 warn
39	18:24:43	38	48	63	1.55	-0.08	31.3	4.99	12	Automatic	pDisp O2 warn
40	18:24:48	38	61	71	1.5	-0.11	31.2	5	5	Automatic	OK
41	18:24:52	38	83	102	1.53	-0.13	29.1	4.99	5	Automatic	OK
42	18:24:57	38	90	123	1.58	-0.14	29	4.99	5	Automatic	OK
43	18:25:01	38	91	136	1.55	-0.15	29	4.99	5	Automatic	OK
[illegible]	[illegible]	[illegible]	[illegible]	[illegible]	[illegible]	[illegible]	[illegible]	[illegible]	[illegible]	[illegible]	[illegible]

Figure S5: Exportable file format for process parameters. Here, the first minutes of the synthesis of $Zn_5Zr_{95}O_y$. from campaign 3 are depicted. The complete collection of syn files can be found in the external data repository. The temperature decreases of T3 and T4 at the beginning of a pyrolysis run are the result of changing airflow patterns as the vacuum pump is ramped up. A few seconds before starting the recording, the airflow was <10 $Nm^3\ h^{-1}$. Some time is required for the sensors to cool down and for the air temperatures to reach an equilibrium, as more air flows through the reactor. Before an equilibrium is reached, injection of the precursor is started. The short peak of "mzr pump ml" in the first few seconds of the recording is a relic of the cleaning procedure of the micro annular gear pump. When starting a new batch, the precursor is pumped towards the waste to remove potential leftover impurities from the hoses and to purge air bubbles. To accelerate this step, the pump rate is increased to the maximum before being adjusted to the setpoint for pyrolysis.

Furthermore, this batch is a good example for the intervention of AutoFSP if process parameters exceed their limits: After about 1 minute, there was a warning for the O_2 dispersion pressure ($pO_{2,\ disp}$). The mzr pump was turned off until the operator corrected the deviation and $pO_{2,\ disp}$ was within the preset range again.

Considerations for the choice of materials used for vessel manufacture

The following materials were considered as candidates for vessel manufacture: stainless steel (SS316), Hastelloy©, titanium, non-elastomeric fluorinated polymers (e.g., massive, lathed PTFE), Polyoxymethylene (POM), and aluminum. Furthermore, additive manufacturing, using metallic or polymeric 3D-printing was considered.

Lathed aluminum was chosen as preferred material for various reasons:

a) It is affordable and widely available with short lead times.
b) It is easy to drill, lathe, cut and hollow out using standard machining tools. Threads are sufficiently strong. Fine surface features, like O-ring-grooves are reliably made.
c) The dosing vessel permanently resides on the precision balance (readability: ± 10mg) and therefore needs to be sufficiently lightweight to not exceed the maximum rated capacity.
d) It is not ferromagnetic, enabling stirring by means of a magnetic stir bar.

Bill of materials

Table S5: Major system components and estimate cost required for the construction of both, AutoFSP and its conventional counterpart. Country Codes: DE = Germany, USA = Unites States of America, CH = Switzerland, FR = France, JAP = Japan, DK = Denmark, CN = China.

Category	*Item*	*Detail*	*Manufacturer*	*Cost per unit [CHF]*	*Units Used*	*Total Cost [CHF]*
Inlet and exhaust-gas manage-ment	Vacuum pump	MINK 0080D, 80 $Nm^3\ h^{-1}$	Busch Vacuum Solutions, Maulburg, DE	4600	1	4600
	Heat exchanger for exhaust gas	GPLK 30-30-H-30	Funke, Gronau/Leine, DE	220	1	220
	Stainless steel exhaust pipe	316L, KF40, with connector s	VACOM, Grosslöbichau, DE	330	3 m	1000
	Pressure sensor Δp_{filter}	PE5 (-1 to 0 MPa-g and 0 to 5 MPa-g)	Emerson Electric Co., USA	150	1	150
	Thermo element	1.5*300		60	4	240
	Inlet airflow hose	Foxi Garant Pur, 45mm	Peter Hefti AG, Frauenfeld, CH	35	1m	35
	Flowmeter inlet airflow	VA520	CS Instruments, Harrislee, DE	1270	1	1270
	HEPA inlet air filter + housing	Megalam MG14 & CamSeal, CSL-3P3-TS-C 160	CAMFIL AG, Oberägeri, CH	500	1	500
Process gas manage-ment	Mass Flow Controllers	Red-y-smart series	Vögtlin Instruments, Muttenz, CH	1483	3	4450
	Push-in fittings & PU-hosing for gases and liquids		Parker Legris	-	-	500
	Pressure sensors, $p_{O2,disp}$	PE5 (0 to 5 MPa-g)	Emerson Electric Co., USA	150	1	150
Micro-annular gear pump	Pump for liquid precursors	mzr-2905	hnp-Mikrosysteme, Schwerin, DE	5400	1	5400

Setup build materials	Nuts, bolts, plates, flanges, strut profile		stocks from inhouse workshop	-	-	3150
Parts for HMI	Computer w/ housing	Raspberry Pi 4	Raspberry Inc.	100	1	100
	12.5” touch screen			180	1	180
PLC Parts	PLC controller	PFC100, 750-8100	WAGO, Minden, DE	382	1	382
	Power supply 24 VCD	750-602		14	2	28
	8-channel Analog In: Thermocouple	750-458		243	1	243
	4-channel analog In, 0-10 VDC	750-459		140	1	140
	RS232/485 Interface	750-652		231	2	462
	4-channel analog Out, 0-10 VDC	750-559		140	1	140
	Housing			140	1	140
	Emergency off switch		Eaton, USA	65	1	65
	Power supply	24VDC, 5A		219	1	219
Various electronics	Plugs, cables, adapters		Various	-	-	680
	Nanoparticle counting devide	AeroTrak 9000	TSI Inc., USA	13165	1	13165
Module Fabrication	Machining Cost	Electronic Workshop	D-CHAB, ETH Zürich, Zürich, CH		90 h	2700
	Machining Cost	Mechanic Workshop			50 h	1500
Total						41’207

Table S6: Additional costs incurred for the conversion from conventional to AutoFSP.

Category	*Item*	*Detail*	*Manufacturer*	*Est. cost unit^{-1} [CHF]*	*Units Used*	*Total Cost [CHF]*
Microfluidic liquid valves w/ FFKM seals	Type 2/2NC	SCS067A031	ASCO Emerson Electric Co., USA	235	10	2350
	Type 3/2	SCS067A111		235	3	705
	Type 3/2, ¼-28UNF	6724	Bürkert, Ingelfingen, DE	190	3	570
	Type 2/2 NC, ¼-28UNF			143	6	860
	Type 2/2NC, R1/8”	6011		157	3	470
Pneumatic Liquid handling	Rotary Valve	PTFE Body, 10-port, 1000 µm Orifice	Advanced Microfluidics, Ecublens, CH.	980	1	980
	Hosing, manifolds & Ferrules	Tubing 1/8”, Ferrules: ¼”-28UNF	Darwin Microfluidics, Paris, FR	-	-	1000
	Pressure Controller 0.05-5 MPa-g,	ITV1030-31F1N3	SMC Corp. JAP	450	1	450
	Type 3/2 gas valves used for vessel pressurization	VUVS	Festo, Esslingen, DE	67	3	200
	Optical bubble sensor	BE-A301P	Panasonic Electronics, JAP	87	4	348
Dosing and Mixing Vessels	Magnetic stirring plates	Rotilabo M3	Roth AG, Arlesheim, CH	100	2	200
	Manufacturing of D&M vessels	Material Cost & Machining	Inhouse workshop	-	-	600
	Coating for D&M vessels	PTFE	Buser Oberflächentechnik, Wiler, CH.	150	3	450
	PTFE Gear Pump	A500S-4NP	Oriwen Fluidics Technology, Shanghai, PRC	400	1	400
	Cleaning Nozzles	1.35-80° H-LE	Danfoss, Nordborg, DK	33	3	100
	Analytical Scale	JJBC-1523	G&G, Kaarst, DE	690	1	690
	Various small parts	Cables, Plugs, Connectors		-	-	500
PLC	Power supply	750-601	WAGO, Minden, DE	17	1	17

	24VCD w/ fuse					
	16-channel digital out, 24VDC	750-1504		100	2	200
	8-channel digital in/out, 24VDC	750-1506		78	1	78
	2-channel relay, NO	750-512		85	1	85
	RS232/485 Interface	750-652		231	1	231
	Power supply, 12VDC, 5A	RND315-00001	RND Power, CN	8	1	8
Manufacturing of Mixing unit housing	Materials, Machining, Anodizing (Al)	Mechanic Workshop	D-CHAB, ETH Zürich, Zürich, CH	-	-	2800
Total						13740

Process times of AutoFSP during production campaign 1

Table S7: Comparison of process times required for a production of identical 8-batch campaigns in conventional and automatic mode.

Working step	*Approximate time requirement [min]*	
	Conventional Operation	Automatic Operation
Preliminary calculations + Planning Synthesis	30	-
Preparing Electronic files with Syn Data	-	5
Diluting and bottling of commercial precursors	20	20
Compounding of precursor mixtures	80 min (10 min per batch)	-
Connection of precursors to manifold	-	5
Readying of the reactor	5	5
Calibration of micro annular gear pump (mzr)	15	5
Compounding + transfer + stirring of batch #1		7
Pyrolysis (5/5 flame condition, 50 mL per batch), incl. filter changes	88 min (@8 batches @ 11min each)	88 min (8 batches @ 11min each)
Time Debit (Operator needs to stay in vicinity of setup, but is free perform other tasks)	-	-40 min (8 batches @ 5min each)
Cleaning of the reactor body	10	10
Cleaning & stowing of materials used (e.g., washing bottles, flushing precursor pump)	30	10
Documentation	20	-
TOTAL	298 min	115 min (155 min w/o time debit)
